# Gossypiboma Following Cesarean Section Presenting as Bilateral Abdominal Abscesses: A Case Report

**DOI:** 10.3390/jcm15062377

**Published:** 2026-03-20

**Authors:** Bogdan-Mihnea Ciuntu, Irina Mihaela Abdulan, Dumitrel Băiceanu, Mihaela Corlade-Andrei, Sorin Nicolae Peiu, Raluca Dragomir, Gheorghe Balan, Andrea Ludușanu, Radu Valentin Averescu, Dan Andronic

**Affiliations:** 1 Faculty of Medicine, Grigore T. Popa University of Medicine and Pharmacy, 16 Universitatii Street, 700115 Iasi, Romania; bogdan-mihnea.ciuntu@umfiasi.ro (B.-M.C.); raluca.dragomir@umfiasi.ro (R.D.); andreealudusanu1106@yahoo.com (A.L.); averescuradu@gmail.com (R.V.A.); candroni@gmail.com (D.A.); 2 General Surgery Clinic, “St. Spiridon” County Emergency Clinical Hospital, 1 Independence Boulevard, 700111 Iasi, Romania; 3 Emergency Care Department, “Sf. Spiridon” County University Emergency Hospital, 700111 Iasi, Romania; mihaela.corlade2@umfiasi.ro; 4 Vascular Surgery Clinic, “Sf. Spiridon” County University Emergency Hospital, 700111 Iasi, Romania; sorin-nicolae.peiu@umfiasi.ro; 5 2nd Gastroenterology Ward, “St. Spiridon” County Hospital, Independence Bvd. No 1, 700111 Iasi, Romania; gheorghe-g-balan@umfiasi.ro

**Keywords:** gossypiboma, computed tomography, abdominal abscess, pseudotumoral mass, retained surgical sponge

## Abstract

**Background**: Gossypiboma is an uncommon postoperative complication caused by the retention of surgical materials, most frequently sponges, and is associated with substantial morbidity and medicolegal consequences. Despite a reduction in reported incidence, diagnosis remains challenging due to its nonspecific clinical presentation. **Case Presentation**: We present the case of a 36-year-old woman who presented with a one-week history of throbbing abdominal pain in the umbilical and left lumbar regions, associated with fetid leukorrhea. Her medical history included an appendectomy, a recent cesarean section performed two months prior, and pregnancy-induced hypertension. Initial computed tomography revealed bilateral subcutaneous and intra-abdominal collections with air bubbles and hyperdense linear structures, raising suspicion of abdominal abscesses. Gynecological evaluation excluded pelvic inflammatory pathology. Exploratory laparotomy identified bilateral pseudotumoral masses with complex adhesions involving intestinal loops and omentum, without evidence of gynecologic infection, prompting transfer to a tertiary care center. Repeat imaging confirmed bilateral flank abscesses. Surgical reintervention revealed retained surgical sponges within both abscess cavities, which were successfully removed, followed by evacuation, lavage, and drainage. Postoperative evolution was favorable under broad-spectrum antibiotic therapy, with complete clinical and biological recovery. **Conclusions**: This case highlights the diagnostic challenge of gossypiboma, particularly when mimicking intra-abdominal abscesses or adhesion syndromes. A high index of suspicion is required in patients with prior surgical history and atypical postoperative presentations, as early recognition and prompt surgical management are essential to reduce morbidity and medicolegal consequences.

## 1. Introduction

Gossypiboma, also known as textiloma, refers to the inadvertent retention of a cotton surgical sponge or compress within the operative field, most commonly in the peritoneal cavity, following surgery [1]. The intraperitoneal space represents the predominant site of gossypiboma localization [1,2]. Although the true incidence remains difficult to determine because of underreporting, a decreasing trend has been observed over recent decades. Earlier estimates reported an incidence of approximately 1 per 1000 abdominal surgical procedures [3], whereas more recent studies indicate a lower rate of 0.08 to 0.18 per 1000 operations [4,5].

The incidence of retained foreign bodies (RFBs) varies by surgical procedure type. Reported identification rates are 17.69% after cesarean section, 16.33% after abdominal hysterectomy, and 13.54% following exploratory laparotomy for acute abdomen [6]. Among all RFBs, gossypibomas account for approximately 17% to 68% of cases [4,5]. In a large series of 4547 cases, Birolini et al. reported that 90% of retained surgical items were textilomas, with large surgical sponges being the most identified materials [6].

Diagnosis may be made early, within days of surgery, or may be delayed for several years, often leading to increased morbidity and, in rare cases, death. Clinical presentation, treatment, and prognosis largely depend on the timing of diagnosis and the tissue response type. Early postoperative symptoms typically include acute inflammatory reactions, such as exudative or purulent processes that can develop into peritonitis or abscesses, accompanied by systemic signs like fever, abdominal pain, nausea, vomiting, abdominal distension, and fatigue. Conversely, delayed cases are usually linked to chronic inflammatory reactions, including xanthogranulomatous inflammation, aseptic fibrosis, and calcifications. In these instances, patients may be symptom-free or experience nonspecific symptoms such as abdominal pain, bowel transit issues, distension, or symptoms related to mass effect [7].

Imaging is essential in diagnosis and includes transvaginal and abdominal ultrasonography, abdominal–pelvic radiography, contrast-enhanced computed tomography, and magnetic resonance imaging [8]. Differential diagnosis should consider other intra-abdominal tumoral lesions [9]. Surgical removal remains the primary treatment in most cases, with increasing technical challenges as diagnosis is delayed. Complications such as perforation, fistula formation, and bowel obstruction often necessitate the involvement of a multidisciplinary surgical team.

Despite the known risk of this adverse event and its significant medicolegal consequences, previous studies have shown that up to 74% of surgeons do not disclose the presence of a retained foreign body to patients, often attributing the need for reintervention to other causes [6].

This article aims to assess the extent and risk of gossypiboma after cesarean section—especially emergency cases—to identify risk factors and help develop strategies to lower its occurrence.

## 2. Case Report

We will discuss the case of a 36-year-old patient who presented to the outpatient clinic at Dorohoi Municipal Hospital with throbbing abdominal pain in the umbilical and left lumbar regions, beginning a week earlier, along with fetid leukorrhea. The patient’s medical history includes an appendectomy at age 21, a cesarean section two months prior to this admission, and pregnancy-induced hypertension.

**Figure 1:**
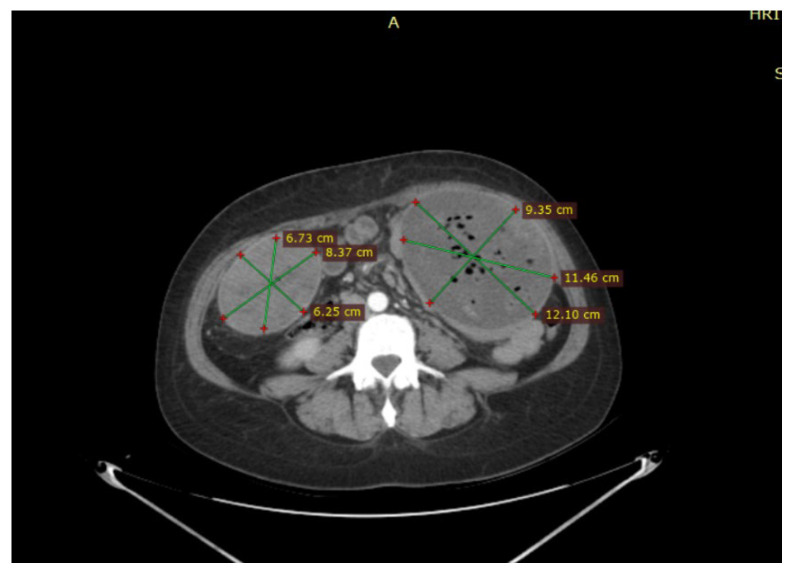
Collections in the left and right subcutaneous flank.

**Figure 2:**
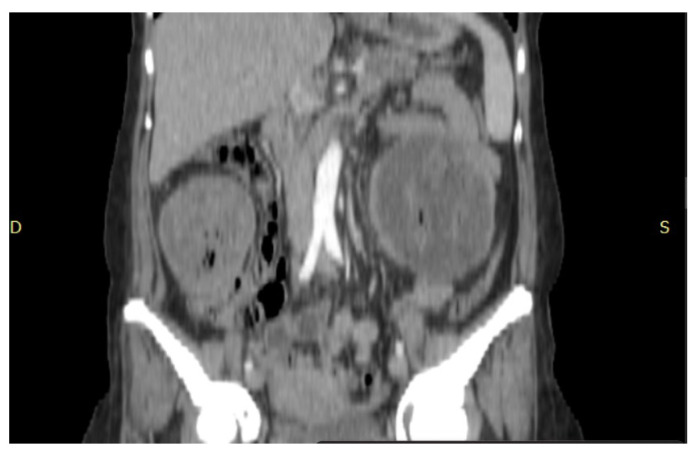
Sagittal view of the collections.

A CT scan performed on admission revealed a collection in the left subcutaneous flank with a relatively thin wall (6 mm), inhomogeneous structure, multiple air bubbles inside, and native hyperdense linear images. It is surrounded by fluid and measures 129/104/141 mm (AP/TR/CC) (Figure 1 and Figure 2), showing contrast uptake at the wall level and causing mass effect on adjacent intestinal loops, with subcutaneous bulging. Immediately anteroinferior to it, near the muscular wall, there is a nodular image with a clear contour, measuring 13/10 mm, with calcified walls. On the right flank, immediately subcutaneous, another collection with a clear contour, a thin wall, and multiple air bubbles inside measures 86/66/94 mm (Figure 1 and Figure 2), also showing contrast uptake at the wall level.

A clinical gynecological examination was performed: visual inspection with specula revealed changes in the vagina, including moderate leukorrhea with a foul odor. Digital vaginal examination showed a cervix with a closed external os, a uterus of normal size that was tender to palpation and movement, non-palpable adnexa, and a free Douglas pouch. In the left hypochondrium, a painful pseudotumoral mass with limited mobility was palpated. Transvaginal ultrasound performed in the outpatient clinic indicated a normal-sized uterus, ovaries with a bilateral follicular appearance, and a small amount of fluid in the Douglas pouch.

The patient was in generally good condition, hemodynamically stable, afebrile, and tachycardic, with pale skin. Cultures were taken from vaginal, pharyngeal, and nasal secretions upon admission. Laboratory results at admission showed a slightly elevated erythrocyte sedimentation rate (ESR) of 35 mm in one hour.

Two days later, a surgical intervention was performed based on the suspected diagnosis of pelviperitonitis and a suspected left flank abscess. Anesthesia consisted of spinal anesthesia supplemented with general anesthesia with orotracheal intubation. Preoperatively, prophylactic Cefort 2 g IV and Metronidazole 1 vial in a single dose were administered. The surgical procedure involved an iterative Pfannenstiel incision over the old scar. Upon opening the peritoneal cavity, the internal genital organs appeared normal. Manual exploration of the abdominal cavity revealed two pseudotumoral formations—one in the left flank and another in the right flank—containing intestinal loops and the greater omentum.

The medical-surgical team decided to continue the surgical intervention with an upper and subumbilical laparotomy. After opening the peritoneal cavity, a manual exploration revealed a large tumor in the left flank, anteriorly fixed to the abdominal wall and posteriorly to the viscera in the supramesocolic region. Similarly, in the right flank and right hypochondrium, a formation adherent to the abdominal wall and posteriorly connected to the liver, colon, and duodenum was identified. It is important to note that the old sclerotic process prevented the release of both formations without major iatrogenic risks that could not be managed locally due to the absence of a specialized surgical team. Consequently, the decision was made to limit the procedure to an exploratory laparotomy, with the patient transferred to a university hospital. A peritoneal fluid culture was obtained, the peritoneal cavity was irrigated, the abdominal wall was reconstructed in anatomical layers, the skin was sutured, and a dressing was applied. The urine through the catheter remained normochromic.

Postoperative diagnosis: complex adhesion syndrome and abdominal pseudotumoral formations. The patient did not exhibit symptoms of metritis across clinical, paraclinical, imaging, and surgical assessments.

The patient was transferred to the St. Spiridon County Emergency Clinical Hospital in good overall condition, afebrile, hemodynamically and respiratory stable, with normal diuresis, minimal serosanguinous drainage, a clean postoperative wound, and a sterile dressing.

After admission to the Surgery Clinic at St. Spiridon County Emergency Hospital, new tests were conducted that showed leukocytosis with neutrophilia, anemia, elevated C-reactive protein (CRP), and slightly increased sodium levels. A new ultrasound was performed, revealing an inhomogeneous transonic formation in the left flank with a wall measuring 150/76 mm (suggestive of an abscess). In the right flank, a similarly characterized formation was identified, measuring 100/80 mm.

**Figure 3:**
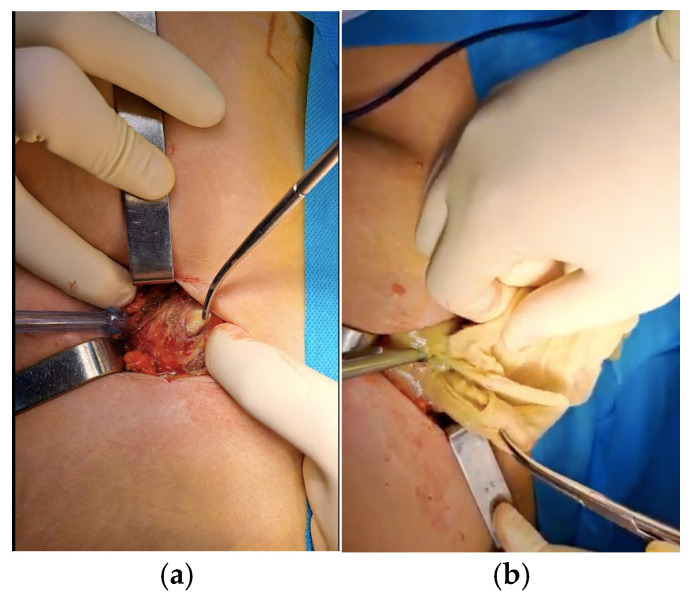
(**a**,**b**)—Intraoperative images capturing the moment of gossypiboma identification and its extraction.

The following day, surgical intervention was repeated by making a paramedian incision in the left flank, dissecting the anatomical planes until entering the peritoneal cavity, then accessing the abscess cavity where a textile foreign body (compress) was identified (Figure 3). The foreign body was removed, samples were collected for cultures and antibiogram, the abscess was drained, lavaged, and a drain was placed. The incision was closed in anatomical planes, skin sutures were applied, and a sterile dressing was placed.

Postoperatively, the biological balance was restored, and the patient received broad-spectrum antibiotic therapy. Hydro-electrolyte and acid-base rebalancing were performed, along with pain management and prophylaxis for deep venous thrombosis and pulmonary thromboembolism.

The patient’s condition improved with the remission of initial symptoms, hemodynamic and respiratory stability, resumption of oral nutrition with good digestive tolerance, intestinal transit present for feces and gases, normal diuresis, minimal drainage, and clean postoperative wounds in the process of healing. The ultrasound examination performed on the third postoperative day showed no right or left pleural fluid, no fluid in the Morrison space or in the pouch of Douglas, a normal-appearing uterus, non-dilated bowel loops, and a 12 mm fluid collection in the right ovary. One of the drainage tubes was removed, and the other was taken out the following day.

On the fifth postoperative day, the patient was discharged with recommendations to avoid intense physical exertion; perform local wound care and use sterile dressings; remove sutures after 21 days; and continue prophylaxis for deep venous thrombosis and pulmonary thromboembolism for another 14 days.

## 3. Discussion

Gossypiboma, also known as textiloma, is a significant medical issue due to its medicolegal implications and the higher risk of patient morbidity and mortality [1]. The true frequency of this complication remains hard to determine because reporting is inconsistent and often incomplete. However, the reported global rate in abdominal surgery ranges from 1 to 1.2 cases per 1000–1500 procedures [9]. In a large study involving 49,831 surgeries performed under general anesthesia, 24 cases of retained foreign bodies were found (0.48 per 1000 surgeries), of which 17% (0.08 per 1000) were gossypibomas [5].

An increased occurrence of gossypiboma has consistently been linked to emergency surgical procedures, especially in obstetric settings such as placenta previa, placenta accreta, severe hemorrhage, and uterine rupture. Emergency interventions are the most common risk factor, making up about 26% of cases, with incorrect sponge counts close behind at 25% [6]. Several factors have been suggested to explain this higher risk, including failure to follow surgical protocols, poor communication and coordination among the surgical team during operations, inadequate staff training, unexpected changes in the surgical plan that require multidisciplinary involvement, inaccurate sponge counts due to heavy blood loss, lengthy and technically complex procedures, intraoperative hemodynamic instability, higher body mass index, and related health conditions [9].

To better characterize gossypibomas after cesarean section, a comprehensive electronic search of the PubMed database was conducted from its inception until March 2026 using the MeSH terms “gossypiboma”, “textiloma”, “gauze”, “sponge”, and “cesarean section”. This search identified a total of 59 published cases.

The average interval between cesarean section and gossypiboma diagnosis was 3.69 ± 6.24 years, ranging from 0.04 to 29 years. This extended diagnostic delay indicates that many cases remain asymptomatic or have few symptoms for an extended period. Similar observations were reported by Birolini et al., who observed asymptomatic and oligosymptomatic cases in 12% and 71%, respectively [6]. The average age at diagnosis was 34.58 ± 8.38 years (range: 20–68 years), which corresponds to the reproductive age group most frequently undergoing cesarean delivery and is an independent risk factor for gossypiboma development.

Although several studies have reported that gossypibomas are most often diagnosed within the first two months after surgery, our analysis showed a diagnostic rate of only 19.29% during this period, with a peak incidence of 47.36% occurring more than a year after surgery. This delayed diagnosis may be due to the relatively low proportion of severe clinical symptoms, reported in only 17% of cases [6]. The body’s response to retained textile material varies depending on how long it remains in place and can involve either an aseptic fibrinous reaction that leads to encapsulation or an exudative inflammatory process that causes abscess formation [7].

The clinical presentation varies based on the location, size, and number of retained sponges, as well as on complications such as subacute intestinal obstruction or peritonitis. Pain is the most common early symptom, often accompanied by a palpable abdominal mass. In contrast, late presentations might be asymptomatic or minimally symptomatic and are often found incidentally years after surgery [6,10,11]. In this case, gossypiboma was diagnosed 16 months after an emergency cesarean supracervical hysterectomy, following the development of chronic lower abdominal pain and a deforming abdominal wall mass.

The natural progression of retained foreign bodies can include fibrotic encapsulation, intraluminal migration into the intestine, vagina, or urinary bladder, and the development of abscesses or fistulas [2]. These processes can significantly hinder imaging diagnosis and may cause diagnostic confusion. The differential diagnosis encompasses cystic or pseudocystic lesions, neoplastic masses, abscesses, hematomas, and granulomatous formations [9].

Ultrasonography is usually the first imaging method used, enabling evaluation of lesion size, structure, blood flow, and relationships with nearby organs. Conventional radiography can detect intestinal obstruction or altered anatomy, but often falls short due to the lack of radiopaque markers.

Contrast-enhanced computed tomography and magnetic resonance imaging greatly improve preoperative diagnosis accuracy and are considered the most effective techniques for gossypiboma detection [8,10,12].

The most frequently reported complications associated with gossypiboma include fistula formation (19.29%), perforation (12.28%), bowel obstruction (5.26%), and urinary bladder injury [10,13,14,15,16,17,18,19,20,21,22]. Management is primarily surgical and depends on the timing of diagnosis and the presence of associated complications. Thorough preoperative evaluation is essential, as fistulas, perforations, abscesses, or extensive adhesion syndromes involving adjacent organs can significantly increase operative complexity, surgical duration, and length of hospital stay.

Late diagnosis usually involves strong inflammatory and granulomatous reactions, resulting in dense adhesions that make surgical removal more difficult. In contrast, early diagnosis is more often linked to acute peritonitis, requiring urgent surgical intervention. Depending on the location and extent of involvement, removing the retained textile material may involve bowel resection with anastomosis, as well as resection of omental, hepatic, gastric, or adnexal structures [1,10,11].

Prevention remains the key to reducing the incidence of gossypiboma and depends mainly on careful counting of all textile materials, thorough examination of the peritoneal cavity before wound closure, and strict adherence to surgical safety protocols. The use of sponges with radiopaque markers or electronic tracking systems can further improve intraoperative detection and decrease the risk of retained foreign bodies [7,10,23].

## 4. Conclusions

Preventing postoperative foreign bodies (e.g., a forgotten compress) relies on organizational, technical, and team communication strategies. These are among the most crucial safety measures in the operating room.

Having clear protocols for emergency situations is crucial, especially in chaotic interventions like postpartum hemorrhage, where the risk is higher. Marking and counting all textile materials must be done carefully, following the inventory protocol strictly. Separate counters (two people) should perform the counting, and wound closure should only occur after a correct and complete inventory.

## Data Availability

The original contributions presented in this study are included in the article. Further inquiries can be directed to the corresponding author.
